# Economic Evaluations of Stepped Models of Care for Depression and Anxiety and Associated Implementation Strategies: A Review of Empiric Studies

**DOI:** 10.5334/ijic.4157

**Published:** 2019-06-21

**Authors:** Penny Reeves, Zoe Szewczyk, Judith Proudfoot, Nyree Gale, Jennifer Nicholas, Josephine Anderson

**Affiliations:** 1 Hunter Medical Research Institute, AU; 2 School of Medicine and Public Health, University of Newcastle, AU; 3 Black Dog Institute, University of New South Wales, Sydney, AU

**Keywords:** stepped care, integrated mental health care, economic evaluation, healthcare decision-making

## Abstract

Since the mid-2000s stepped care, a model of integrated healthcare delivery, has been promoted for offering accessible, effective and efficient services for individuals with mental health conditions. However, adoption of the model has not been widespread warranting additional investment by way of implementation strategies to encourage uptake. These strategies also require funding and their value for money should be assessed to inform decision making and practice. We conducted a review to better understand the extent to which the cost-effectiveness of stepped care has been evaluated (review i) and also to investigate whether economic evaluation has been applied to implementation strategies of stepped care services for anxiety and depression in developed (high income) countries and to chart their methods and outcomes (review ii). The searches were conducted in six electronic databases, grey literature and relevant journals. The search strategies returned two papers for reviews (i) and a single paper for review (ii). Despite stepped care models of integrated mental health service provision being promoted as optimal, there is limited knowledge surrounding the real-world cost-effectiveness of their application and clearly a need for good quality economic evaluations of integrated care that comply with international guidelines of good practice. There is even less information pertaining to the cost-effectiveness and budget impact of strategies designed to increase the uptake of these models.

## Introduction

Since the early 2000s there has been recognition that improving the detection, treatment and outcomes for mental health problems requires service models that integrate mental health care within primary health care practice [1,2]. More recently, online or web-based mental health interventions have been shown to have an important role in the provision of mental health care [3,4]. A common framework used to integrate efficacious and efficient web-based mental health interventions into primary care settings is stepped care. Stepped care is a model of integrated healthcare delivery that claims to offer accessible, effective and efficient services for individuals with mental health difficulties [5]. The stepped care model has two key features: First, the recommended treatment for a person is the least restrictive available, meaning that the impact of the treatment on patients with respect to both cost and inconvenience is minimised but the treatment mode is still likely to produce health gain [6]. In stepped care, more intensive treatments are generally reserved for people who do not benefit from simpler, first-line treatments or for those who can be accurately predicted not to benefit from such treatments [7]. Second, the model is “self-correcting” [8]. That means the least intensive intervention appropriate for a person is typically provided first and with regular monitoring, more or less intensive treatments are subsequently provided according to a person’s changing needs and response to treatment. The approach to stepped care interventions can be ‘progressive’ whereby all patients commence at step one, with subsequent interventions provided to those who require it; or ‘stratified’ where patients are provided the least intensive intervention matching the severity of their symptoms and available resources [9,10]. The importance and expected efficiency of the stepped care model has gained prominence both internationally and in Australia [11,12,13]. Yet, despite strong recommendations by government [11] and practitioners [14], a stepped care mental health service that is fully articulated and integrated into primary care is yet to be implemented in Australia.

Successful implementation of such a complex mental health treatment model requires an understanding of personal, organisational and systemic factors that influence the success or failure of integration. Implementation science as a field has evolved to better understand these barriers and facilitators to increasing uptake of effective and cost-effective interventions. Implementation strategies such as education and training of healthcare providers and financial incentives are examples of mechanisms to change behaviour. However, demonstrating effectiveness and cost-effectiveness is a necessary but, on its own, insufficient factor to promote translation and scalability. For practitioners, understanding relative effectiveness of interventions is critical. However, for policy decision making the extra step of understanding the relative cost of alternative pathways is needed. Understanding cost reveals the extent to which value is being delivered from a model of care. There is also a need to place the evidence in a format decision makers understand, hence budget impact statements, that highlight relevant effects and costs, are warranted [15].

In this study, we reviewed the published literature for evidence pertaining to both the cost-effectiveness of stepped care models adopted in developed (high income) countries and also the cost-effectiveness of implementation strategies designed to achieve greater adoption of stepped care. Specifically, our aims were to: (1) identify empirical economic evaluations of stepped care and associated implementation models published since 2000; (2) assess the quality of these economic evaluations; and (3) synthesise the evidence of the study findings. As described by Bower et al. there is a paucity of evidence supporting the efficiency claim associated with stepped care and a need to quantify the overall public health benefit of traditional and stepped care models [8]. Implementation studies were included in the review for the reason that their cost and cost effectiveness is often overlooked [16,17]. Managing per capita expenditure on health and pursuing maximal health outcomes are dual aims that dominate the health policy agenda of developed countries. It is therefore imperative to have robust evidence of both the effectiveness and cost of technologies introduced to the health system, including models of care.

## Methods

### Search strategies and inclusion criteria

The following databases were searched for English language publications between January 2000 and December 2017: Medline, Embase, PsycINFO, EconLit, the Cochrane Library and NHS EED. Grey literature was hand searched in Google and Google scholar. This timeframe was considered appropriate since the modern configuration of stepped care models emerged with the advent of online self-help services. The reviews were conducted separately for each of the themes (i) economic evaluations of stepped care models and (ii) economic evaluations of implementation strategies targeting stepped care. Details of the Medline search strategies for each review are provided in the supplementary material.

**Figure 1:**
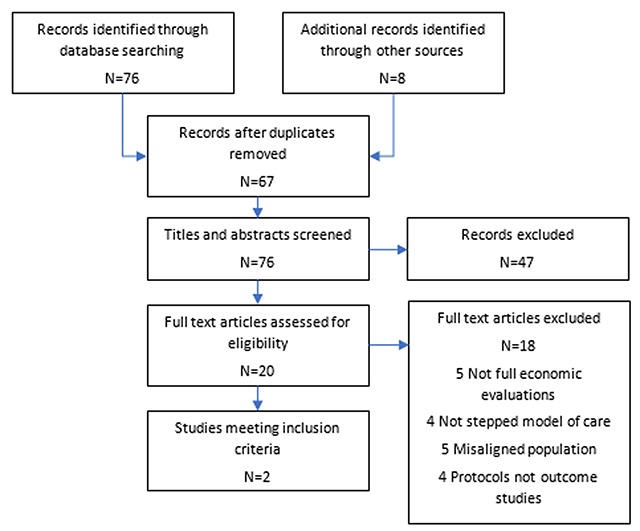
Prisma diagram review (i).

**Figure 2:**
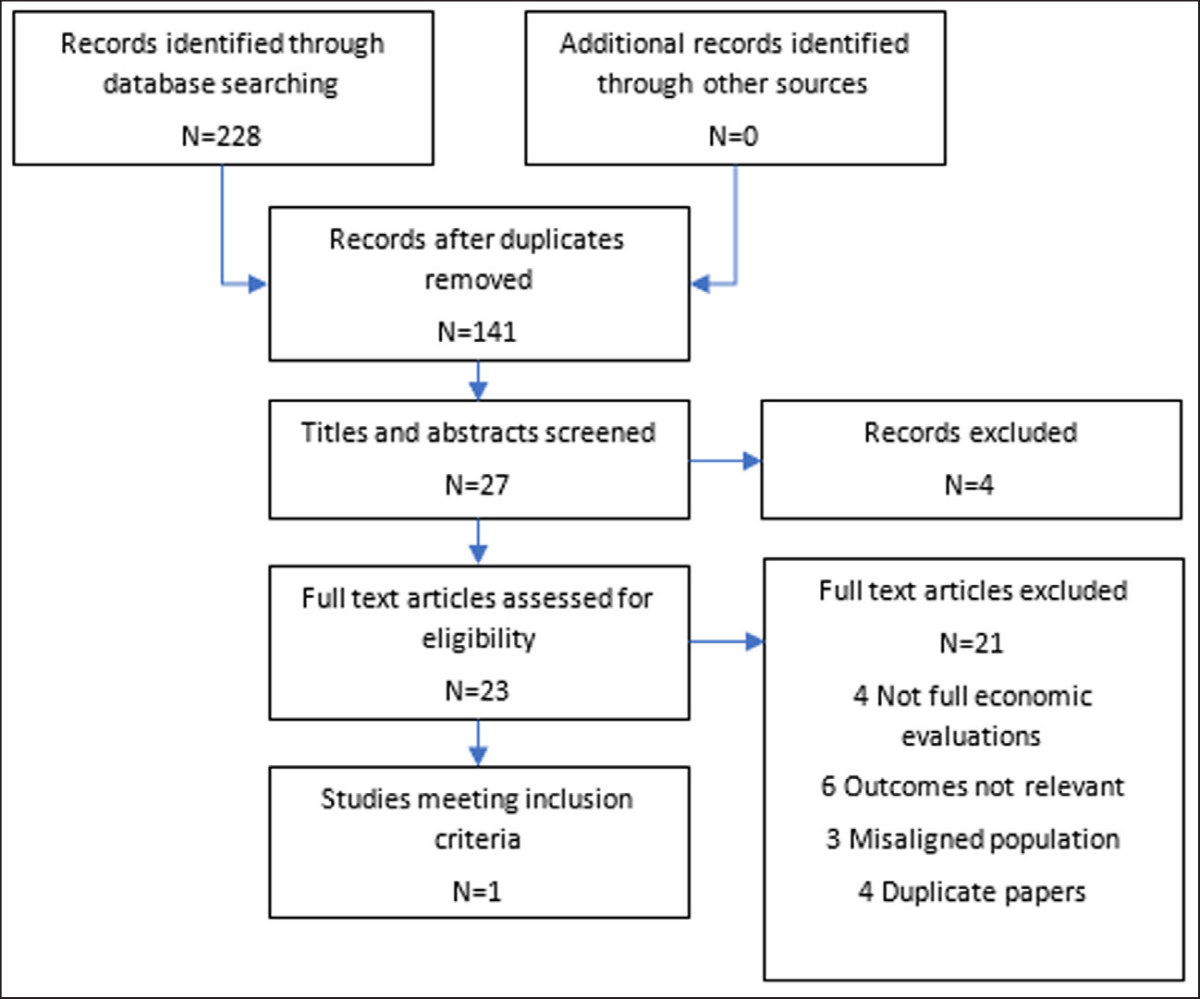
Prisma diagram review (ii).

An iterative approach to the reviews was used. First, an initial search of Medline via the OVID platform was conducted. This was followed by analysis of the text words contained in i) the title ii) the abstract and iii) index terms used to describe the resultant articles. A second search using all identified keywords and index terms was then undertaken in all included databases. Reference lists from all identified reports and articles were hand searched for additional studies and where necessary, authors were contacted for further information. Records were stored and managed in Endnote reference management software. The review process was undertaken independently by two individuals (ZS and PR). Both reviews followed the internationally recognised PRISMA protocol for the assessment of literature [18], Figures 1 and 2.

Included studies were randomised controlled trials, controlled trials, controlled before and-after studies that involved economic evaluations, including modelled evaluations. For review (i) we included all studies that involved full economic evaluations of stepped models of care applied to common mental health disorders – anxiety and depression, in a primary care setting. The choice of inclusion criteria was governed by Australian government policy directing the reform of primary mental health care, with programs covering “Promotion, prevention and early intervention” comprising “efforts to reduce the impact of depression and anxiety” [11].

All studies that involved stepped models of care were included, irrespective of the mode of the first step and mode and intensity of subsequent steps. The primary care setting was specifically selected due to the emphasis in government policy guidance [13,19]. Studies were considered full economic evaluations only if they reported information on both the costs and effects of the intervention [20,21]. For review (ii) we included all studies that involved full economic evaluations of implementation strategies directed towards improved uptake of stepped models of care. The type of implementation intervention could include any or all initiatives designed to influence the uptake of stepped models of care. Examples of implementation strategies include audit and feedback, education, financial incentives and regulation.

### Data extraction and analysis

Two reviewers (ZS and PR) independently extracted data on the study settings and participants, targeted behaviors, model of stepped care, guidelines and strategies, quality of study design, and methods for economic evaluation. The Consolidated Health Economic Evaluation Reporting Standards (CHEERS) checklist was used as the data extraction tool [21].

The quality of the economic studies was assessed using the Drummond 10-point checklist [22]. Reporting quality was assessed using the CHEERS checklist [21].

## Results

### Review (i) Economic evaluations of stepped models of care for mental health disorders

The strategy for search (i) identified 76 citations with an additional 8 records identified through other sources (see PRISMA diagram, Figure 1). After removal of duplicates, 67 articles were screened based on title and subsequently the abstracts of 41 papers were retrieved. Three articles were immediately excluded as two were not available in English and one was published anonymously. Following abstract screening 20 papers remained for full text screening. Of the papers excluded at the full text screening stage, five papers did not include full economic evaluations, four papers did not involve a stepped care model, five papers were not the relevant population and an additional four papers were protocols. Two papers, Goorden et al. [23] and van der Aa [24] strictly met the inclusion criteria. Table 1 below presents a summary of the study characteristics for each of the two included studies.

**Table 1:** Review (i) study characteristics.

	Goorden, M, Muntingh, A, van Marwijk, H, et al.	van der Aa, HPA, van Rens, GHMB, Bosmans, JE, Comijs, HC, van Nispen, RMA

*Jurisdiction*	Netherlands	Netherlands
*Publication year*	2014	2017
*Population & setting*	People with panic and generalised anxiety disorders in primary care	Depression and anxiety in older adults with vision impairment
*Study design*	Trial-based economic analysis of a 2-armed cluster, randomised trial	Trial-based analysis of a multi-centre randomised controlled trial
*Sample size*	43 primary care practices; 180 patients (n = 114, SC and n = 66 TAU)	265 older adults (n = 131, intervention. N = 134, TAU)
*Economic analysis*	Cost utility analysis	Cost utility analysis/cost effectiveness analysis
*Model of stepped care*	Progressive	Progressive
*Comparison*	Treatment as usual	Treatment as usual
*Perspective*	Societal	Societal
*Time horizon*	12 months	24 months

Adherence to the CHEERS reporting guidelines for economic evaluations was high for both of the included studies (Table 3). Primary and secondary outcomes were clearly outlined and there was sufficient detail describing the methods for measuring and valuing effects, including the source of empirical valuations of the health descriptions in the EQ-5D, to generate utilities. Similarly, there was sufficient detail describing the methods for measuring and valuing costs, both direct medical and indirect non-medical costs, including lost productivity.

In the study by Goorden et al. the cost-utility of a collaborative stepped care intervention for panic disorder and generalised anxiety disorder in primary care (GP clinics) was compared to treatment as usual (TAU) from a societal perspective. The choice of perspective is important and in this study all relevant costs to society associated with the burden of anxiety disorders were taken into account, including costs attributable to contact with health care providers, direct medical costs and productivity costs. The intervention consisted of four integrated evidence-based treatment steps: guided self-help, cognitive behavioural therapy, antidepressants according to a medication algorithm and optimization of medication in primary care or referral to secondary care. Cost-utility was calculated by relating the difference in direct medical costs per patient receiving stepped care or TAU to the difference in quality adjusted life years gained, resulting in a cost per QALY estimate.

The economic evaluation based only on direct medical costs, produced an incremental cost-effectiveness ratio (ICER) in the northeast quadrant of the cost-effectiveness plane. That is, stepped care was associated with higher costs and a gain in QALYs. The calculated ICER of 6,965 Euros was reasoned to be significantly less than an upper willingness to pay threshold of 80,000 Euros. The expansion of the analysis to include societal costs showed that collaborative stepped care dominated treatment as usual. That is, stepped care was both more effective and cost less than TAU justifying the claim that collaborative care is a highly cost-effective intervention. Contrary to the position that stepped care is less expensive than TAU, this study showed that quality of life and productivity gains were the main efficiency drivers.

The study by van der Aa et al. [24], describing a trial-based economic evaluation, also reported that a stepped model of care was dominant to usual care (UC) The reported evaluation methods were highly transparent, including those used to measure and value both effects and costs. Similar to the Goorden study, a societal perspective was selected. Overall, the economic evaluation showed that stepped-care was dominant to usual care, that is, more effective and less costly. However, a significant difference in the incidence of depressive/anxiety disorders and symptoms of anxiety did not translate into a difference in QALYs. In contrast to the Goorden study, direct healthcare costs were lower for the stepped-care group driven by significantly lower secondary mental healthcare and hospitalisation costs.

### Review (ii) Economic evaluations of implementation strategies

The strategy for review (ii) identified 228 citations with no additional records identified through other sources (see PRISMA diagram, Figure 2). After removal of duplicates, 141 articles were screened based on title and subsequently the abstracts of 27 papers were retrieved. Following abstract screening 23 papers remained for full text screening, of which four did not include full economic evaluations, three were not in a relevant population, five measured the wrong outcomes for this review, five were protocols and four were duplicate papers. One study strictly met the inclusion criteria [25,26]. Table 2 below presents a summary of the study characteristics for the single study strictly meeting the inclusion criteria.

**Table 2:** Review (ii) study characteristics.

	Sinnema, H, Majo, MC, Volker, D, et al.

*Country*	Netherlands
*Publication year*	2015 (Protocol 2011)
*Population & setting*	General practitioners (including solo practices, group practices or health centres)
*Study design*	Cluster randomised controlled trial
*Sample size*	46 GPs from 23 practices (n = 12, intervention. N = 11, control). 444 patients (n = 198, intervention. N = 246, control).
*Economic analysis*	Cost effectiveness
*Comparison*	Training and feedback pertaining to the recognition, diagnosis, stepped treatment and patient education about anxiety and depression
*Implementation strategies*	Training & feedback as per control plus interventions that were tailored to prospectively identify local barriers. To provide insight into the perceived barriers to early recognition of anxiety and depression, appropriate diagnosis, appropriate treatment allocation and patient education.
*Perspective*	Societal
*Time horizon*	12 months

Adherence to the CHEERS reporting guidelines for economic evaluations was moderate for the study by Sinnema et al. (Table 3).

**Table 3:** Adherence to CHEERS reporting guidelines.

CHEERS Section/item	Item No	Recommendation	Goorden, M, et al. (2014)	van der Aa, HPA, et al. (2017)	Sinnema, H, et al. (2015)

Title	1	Identify the study as an economic evaluation or use more specific terms such as “cost-effectiveness analysis”, and describe the interventions compared.	✓	✓	✓
Abstract	2	Provide a structured summary of objectives, perspective, setting, methods (including study design and inputs), results (including base case and uncertainty analyses), and conclusions.	✓	✓	✓
Background and objectives	3a	Provide an explicit statement of the broader context for the study.	✓	✓	✓
	3b	Present the study question and its relevance for health policy or practice decisions.	✓	Partial	Partial
Target population and subgroups	4	Describe characteristics of the base case population and subgroups analysed, including why they were chosen.	Partial	✓	✓
Setting and location	5	State relevant aspects of the system(s) in which the decision(s) need(s) to be made.	⨯	⨯	⨯
Study perspective	6	Describe the perspective of the study and relate this to the costs being evaluated.	✓	✓	Partial
Comparators	7	Describe the interventions or strategies being compared and state why they were chosen.	✓	✓	✓
Time horizon	8	State the time horizon(s) over which costs and consequences are being evaluated and say why appropriate.	Partial	Partial	Partial
Discount rate	9	Report the choice of discount rate(s) used for costs and outcomes and say why appropriate.	NA	⨯	NA
Choice of health outcomes	10	Describe what outcomes were used as the measure(s) of benefit in the evaluation and their relevance for the type of analysis performed.	Partial	✓	Partial
Measurement of effectiveness	11a	*Single study-based estimates*: Describe fully the design features of the single effectiveness study and why the single study was a sufficient source of clinical effectiveness data.	Partial	Partial	Partial
	11b	*Synthesis-based estimates*: Describe fully the methods used for identification of included studies and synthesis of clinical effectiveness data.	NA	NA	NA
Measurement and valuation of preference based outcomes	12	If applicable, describe the population and methods used to elicit preferences for outcomes.	✓	✓	NA
Estimating resources and costs	13a	*Single study-based economic evaluation:* Describe approaches used to estimate resource use associated with the alternative interventions. Describe primary or secondary research methods for valuing each resource item in terms of its unit cost. Describe any adjustments made to approximate to opportunity costs.	✓	✓	✓
	13b	*Model-based economic evaluation:* Describe approaches and data sources used to estimate resource use associated with model health states. Describe primary or secondary research methods for valuing each resource item in terms of its unit cost. Describe any adjustments made to approximate to opportunity costs.	NA	NA	NA
Currency, price date, and conversion	14	Report the dates of the estimated resource quantities and unit costs. Describe methods for adjusting estimated unit costs to the year of reported costs if necessary. Describe methods for converting costs into a common currency base and the exchange rate.	Partial	Partial	Partial
Choice of model	15	Describe and give reasons for the specific type of decision-analytical model used. Providing a figure to show model structure is strongly recommended.	NA	NA	NA
Assumptions	16	Describe all structural or other assumptions underpinning the decision-analytical model.	NA	NA	NA
Analytical methods	17	Describe all analytical methods supporting the evaluation. This could include methods for dealing with skewed, missing, or censored data; extrapolation methods; methods for pooling data; approaches to validate or make adjustments (such as half cycle corrections) to a model; and methods for handling population heterogeneity and uncertainty.	Partial	✓	✓
		Report the values, ranges, references, and, if used, probability distributions for all parameters. Report reasons or sources for distributions used to represent uncertainty where appropriate. Providing a table to show the input values is strongly recommended.			
Study parameters	18	For each intervention, report mean values for the main categories of estimated costs and outcomes of interest, as well as mean differences between the comparator groups. If applicable, report incremental cost-effectiveness ratios.	⨯	Partial	Partial
Incremental costs and outcomes	19	*Single study-based economic evaluation*: Describe the effects of sampling uncertainty for the estimated incremental cost and incremental effectiveness parameters, together with the impact of methodological assumptions (such as discount rate, study perspective).	Partial	✓	✓
Characterising uncertainty	20a	*Model-based economic evaluation*: Describe the effects on the results of uncertainty for all input parameters, and uncertainty related to the structure of the model and assumptions.	Partial	Partial	Partial
	20b	If applicable, report differences in costs, outcomes, or cost-effectiveness that can be explained by variations between subgroups of patients with different baseline characteristics or other observed variability in effects that are not reducible by more information.	NA	NA	NA
Characterising heterogeneity	21	Summarise key study findings and describe how they support the conclusions reached. Discuss limitations and the generalisability of the findings and how the findings fit with current knowledge.	⨯	Partial	Partial
Study findings, limitations, generalisability, and current knowledge	22	Describe how the study was funded and the role of the funder in the identification, design, conduct, and reporting of the analysis. Describe other non-monetary sources of support.	✓	✓	Partial
Source of funding	23	Describe any potential for conflict of interest of study contributors in accordance with journal policy. In the absence of a journal policy, we recommend authors comply with International Committee of Medical Journal Editors recommendations.	✓	✓	✓
Conflicts of interest	24	Identify the study as an economic evaluation or use more specific terms such as “cost-effectiveness analysis”, and describe the interventions compared.	✓	✓	✓

NA: Not applicable.

The two papers by Sinnema et al. included a study protocol [25] and corresponding outcomes paper [26]. The protocol outlined the design of a study examining whether tailored guideline implementation strategies, supplemented with training and feedback, is more effective than providing training and feedback alone. The outcomes paper presented the results of the study including a cost effectiveness analysis published as supplementary material (additional file 2). Cost-effectiveness was determined by calculating the cost of medical treatment and costs associated with loss of productivity. The incremental cost was reported to be 6,807 Euros to each additional recognised patient in the intervention group. However, the economic outcome is incorrectly interpreted in the paper. The outcome was calculated as the ratio of the difference in cost divided by the difference in outcome (proportion of patients with adequately recognised and documented anxiety or depression). This ICER calculation produces an incremental cost per percent increase in recognised patients.

## Discussion

In this paper we present the results from two literature reviews conducted to better understand the evidence base pertaining to the cost-effectiveness of integrated or collaborative stepped models of mental health care as well as the cost-effectiveness of implementation strategies designed to achieve greater uptake of these models. For the period January 2000 to December 2017, we identified only three empirical studies that strictly met the inclusion criteria reporting both costs and measures of effect [23,24,26]. The limited economic evidence highlighted by this finding is a concern but is not surprising. Health economic evaluation applied to specific clinical interventions became common as a result of individual country reimbursement agencies requiring evidence of cost-effectiveness. In Australia formal evaluation, including economic evaluation, of models of care are not routinely conducted, largely because this investment occurs at the local health service level with less evaluation capability and capacity resources to expend.

While the two studies evaluating the cost-effectiveness of stepped models of care were of good quality and were adherent to the CHEERS reporting guidelines, it is interesting to note that the models of stepped care evaluated in these studies did not include web-based guided support or e-CBT as an initial step. It has been argued that internet-based interventions included in stepped models of care, at least theoretically, improve the efficiency of these models [3]. The results from this literature review are too uncertain to be able to support or dispute this claim.

In contrast to the quality of the economic evaluations of stepped models of care, the implementation evaluations were of lower quality and had poorer adherence to the CHEERS reporting guidelines. The findings from the implementation review are comparable to the work of Hoomans et al. who assessed the empirical literature on studies that evaluated the costs and effects of guideline implementation strategies published since 1998 [16]. Based on their results, the authors concluded that the included studies generally lacked methodological rigour and were of limited use in decision-making. Again, the application of economic evaluation to implementation investment is not commonplace. Clearly, given the prominence and policy direction in favour of stepped care, there is a significant need for more evidence regarding the cost-effectiveness of stepped care and associated implementation strategies from the perspective of both the healthcare system and other relevant stakeholders. For policy development and implementation this significant gap in the evidence base poses a challenge. Internationally, the stepped care model is fundamental to mental health care provision [13,27,28]. In Australia, stepped care is at the core of the federal government’s mental health reform agenda and Primary Health Networks are required to incorporate stepped care in their planning and commissioning [19]. However, it is also government policy to manage escalating expenditure on health care. Ensuring the best ‘value for money’ from health expenditure depends on patients receiving the most cost effective services for any given level of funding. For this reason, it is important to assess the real world cost-effectiveness of new models of care and of the implementation strategies designed to increase the uptake of these models.

Understanding the cost-effectiveness profile of the stepped care intervention is important for informing the budget that is available for implementation so that the intervention combined with implementation remain, as a package, cost-effective. This evidence needs to be collated for decision makers so that the immediate and downstream consequence for the healthcare budget is transparent. Decisions that are not based on this information risk adding to the collection of low value or even harmful models of care that need to be removed from healthcare systems. It is therefore imperative that good quality economic evaluations are applied to integrated models of care that comply with international guidelines of good practice.

Additionally, there are important implications stemming from the cost-effective implementation of stepped care for clinical practice. Namely, clinicians are assisted to detect more cases of mental disorder and to better target treatments for individual patients. Matching of treatments with patients’ presenting problems and alignment of services could be expected to reduce the burden on already stretched mental health services, assist doctors’ workload and facilitate better workforce participation. For the patients, appropriate treatment selection would improve clinical outcomes and improve their wellbeing.

The main limitations of our research are firstly, that we did not conduct formal systematic reviews. Secondly, the reviews were constrained in terms of study populations. These reviews were conducted as precursors to conducting an economic analysis of an implementation strategy designed to influence the adoption of stepped care in the Australian primary care system. However, we adopted a systematic approach using multiple reviewers, pre-specified inclusion criteria and screening using the PRISMA model and we determined that the dearth of evidence returned by the reviews warranted presentation in their own right. We conducted a subsequent scan of the literature, broadened to the application of stepped care in other clinical areas, to inform the external validity of our results showed that the application of economic evaluation is also lacking.

We have highlighted the extent to which further research is warranted to improve both the quantity and also the quality of economic evaluations of integrated stepped models of care and associated implementation strategies. The evidence, as it stands, has important gaps that make it of limited value to decision makers. These gaps can be closed by incorporating health economic principles and methods in the evaluation of implementation strategies designed to impact on the uptake of stepped care models.

## Additional Files

The additional files for this article can be found as follows:

10.5334/aogh.4157.s1 Medline Search Review 1. Scoping review of economic evaluations of Stepped care services for anxiety and depression.

10.5334/aogh.4157.s1 Medline Search Review 2. Scoping review of economic evaluations of StepCare implementation strategies for anxiety and depression.
